# The pupil near response is short lasting and intact in virtual reality head mounted displays

**DOI:** 10.16910/jemr.15.3.6

**Published:** 2023-03-27

**Authors:** Hidde Pielage, Adriana A. Zekveld, Sjors van de Ven, Sophia E. Kramer, Marnix Naber

**Affiliations:** Amsterdam UMC, location VUmc, Netherlands; Utrecht University, Netherlands

**Keywords:** Pupillometry, virtual reality, head-mounted display, pupil near response, vergence-accommodation conflict, eye tracking, gaze, vergence

## Abstract

The pupil of the eye constricts when moving focus from an object further away to an object closer by. This is called the pupil near response, which typically occurs together with accommodation and vergence responses. When immersed in virtual reality mediated through a head-mounted display, this triad is disrupted by the vergence-accommodation conflict. However, it is not yet clear if the disruption also affects the pupil near response. Two experiments were performed to assess this. The first experiment had participants follow a target that first appeared at a far position and then moved to either a near position (far-to-near; FN) or to another far position (far-to-far; FF). The second experiment had participants follow a target that jumped between five positions, which was repeated at several distances. Experiment 1 showed a greater pupil constriction amplitude for FN trials, compared to FF trials, suggesting that the pupil near response is intact in head-mounted display mediated virtual reality. Experiment 2 did not find that average pupil dilation differed when fixating targets at different distances, suggesting that the pupil near response is transient and does not result in sustained pupil size changes.

## Introduction

It is well known that the pupils of the eye change their size in response to variations in brightness (pupil light response). In darker conditions the pupils dilate to allow more light to fall onto the retina, whereas in lighter conditions the pupils constrict for the opposite effect (20). However, pupils also change in size when shifting gaze between objects positioned at different distances from the observer (3; 7; for a review on pupillary reflexes, see: 25). For example, shifting gaze from a person standing a few meters away to a handheld phone causes a constriction of the pupil. This has been suggested to control depth of field - i.e. the range of distances at which objects are perceived to be in focus (27). This pupil constriction does not occur in isolation, but as part of a triad of eye related events. This triad consists of: a) a change in tone of the muscles that shape the lenses (accommodation), b) the eyes rotating inwards (vergence) and c) constriction of the pupils (pupil near response) (2; 6). In head mounted displays (HMDs) used for virtual reality (VR) immersion, vergence occurs as normal, but accommodation is disrupted (8). It is not yet clear if the pupil near response persists in HMD-mediated VR, regardless of the disrupted accommodative response.

Let us elaborate on the uncoupling of vergence and accommodation. To focus on an object in real-life, the eyes converge on (i.e. rotate toward) that object, which is coupled to accommodation of the eyes’ lenses. However, a position change of an object evokes uncoupled vergence and accommodation responses in HMD-mediated VR; vergence responses are similar to those in real-life circumstances, but accommodation in HMDs remains constant as the eyes accommodate to the unchanging distance of the physical surface of the HMD (8). It is still unclear what happens to the pupil near response in HMDs (15; 24). A recent study that manipulated convergence and accommodation through prisms and glasses, found that accommodation alone was not sufficient to induce the pupil near response, but vergence was (5). As vergence is intact in HMD-mediated VR, these results raise the expectation that the pupil near response also occurs in HMD-mediated VR, independent of the vergence-accommodation conflict. However, there is not yet convincing evidence from studies using HMDs to support this.

To our knowledge, only a single experiment has attempted to research if target distance influences pupil size when using HMDs (9). In that experiment, participants were asked to sequentially focus several targets at a range of distances while their pupil size was measured. However, the experiment used targets that were relatively dark, as compared to the background. As a result, local luminance was not properly controlled for as differences in perceived luminance could have been evoked by changes in target size (relative to the visual field) when moving to and from the participant. When the dark targets moved closer to the observer, they occupied a larger area of the visual field, darkening retinal illumination and thus dilating the pupils. While Iskander, et al. (9) reported no measurable change in illumination globally (i.e., in the entire display), both local target luminance and the degree of attention for the stimuli likely evoked pupil light responses. Pupil size is known to change with attention shifts (covertly and/or overtly) to dark or bright objects, despite constant overall retinal illumination (4; 13; 17; 23). Closer, and therefore larger, targets draw more attention, which boosts the subjective experience of illuminance (1; 16; 25). This phenomenon causes the pupil to dilate when dark targets move closer and constrict when dark targets move further away. Possibly this confound explains the reversed pupil near response that Iskander and colleagues reported.

The current paper describes two experiments exploring if the pupil near response is present in HMD-mediated VR while strictly controlling for stimulus size and thus illumination. The first experiment was somewhat in line with that of Feil, et al. (5) in that participants made gaze shifts between far and near targets. Event-related pupil responses were compared between trials where a target object would move from a far position to a near position, and trials where the object would move from a far position to another far position at the same distance. It was hypothesized that pupils would constrict more in response to a gaze shift from far-to-near, as compared to a gaze shift from far-to-far. Furthermore, in half of the trials the size of the target was corrected for its distance in order to control for illumination and assess if this affected the pupil near response.

The first experiment used a methodology which could detect pupil near responses when shifting gaze from a far to a near target (3; 5; 7), but it could not be used to replicate the findings by Iskander, et al. (9), who looked at average pupil size while fixating gaze on targets at various distances. Therefore, a second experiment was added, more in line with Iskander, et al. (9). In this experiment, average pupil size was compared between conditions where participants fixated a target placed at different distances. Like in experiment 1, target size was corrected in half of the trials. Following what is known about typical pupil near responses, but in contrast to Iskander et al., (9), it was hypothesized that the fixation of near targets, as compared to far targets, would be associated with more constricted pupils. We hypothesized that this relationship would be inversed when target size was not controlled for.

## Methods

### Participants

A total of 29 participants (19 females, 10 males) aged 19 to 40 years (mean 25.9 years) were recruited to partake in this study, consisting of 2 experiments. All participants self-reported normal or corrected-to-normal (by means of contact lenses) eyesight and no history of eye related diseases, neurological disorders or diabetes. Besides this experiment, participants also took part in another experiment that researched speech-in-noise perception (data reported elsewhere). Participants received 15 euros compensation for participating in both studies. Approval for this study was granted by the medical ethical research committee of the Amsterdam University Medical Center, location VUmc under reference number 2018.308.

### Materials

This study used a HTC Vive Pro Eye HMD with a built-in Tobii eye tracker. The HMD was connected to a high-end desktop computer with a NVIDIA GeForce RTX2080 Super 8GB graphical card and an Intel Core i7-10700K 3.8GHz 8C 125W motherboard, which ran on Windows 10. The experiments were created in the Unity 3D game engine and ran using custom C# scripts. In order to enable VR in Unity, SteamVR software had to be installed as well. To extract data from the eye tracker, two software development kits (SDKs) were used, namely: ‘SRanipal’ and ‘TobiiXR’. Default settings of both SDKs were used.

### Procedure

First, participants were fitted with the HMD. The built-in eye tracker was calibrated using SteamVR’s default calibration software, which asked participants follow a dot that would move after they had fixated it. The dot first appeared at the centre of the HMD and then moved between the corners of an invisible rectangle. Calibration was performed to optimize the performance of the TobiiXR SDK. The HMD displayed a simple virtual environment which consisted of an icosphere (subdivision level 2) with a diameter of 24m. Thin guidelines along the edges of the icosphere were visible, which were intended as a reference point in space to aid depth perception. Next, participants were asked to complete a heterochromatic flicker fusion test (10). A 1.2 m by 1.2 m rectangle appeared at a distance of 1.8 m from the participants whose colour rapidly alternated between the colour of the background (hue: 180°, saturation: 60%, luminance value: 40%) and a red colour (hue: 0°, saturation: 60%, starting luminance value: 40%). Participants were provided a computer mouse and could adjust the luminance value of the red colour using the scrolling wheel. They were asked to find the luminance value at which the flickering of the colours was experienced to be the least intense, which signals equiluminance between the two colours. This was done once. The resulting red colour was used to fill target objects for the remainder of the experiment. Note that shadows and other light related distance cues were disabled for the entire scene to avoid that they could affect luminance. After completing the flicker fusion test, participants moved on to the two experiments.

### Experiment 1

**Figure 1.**
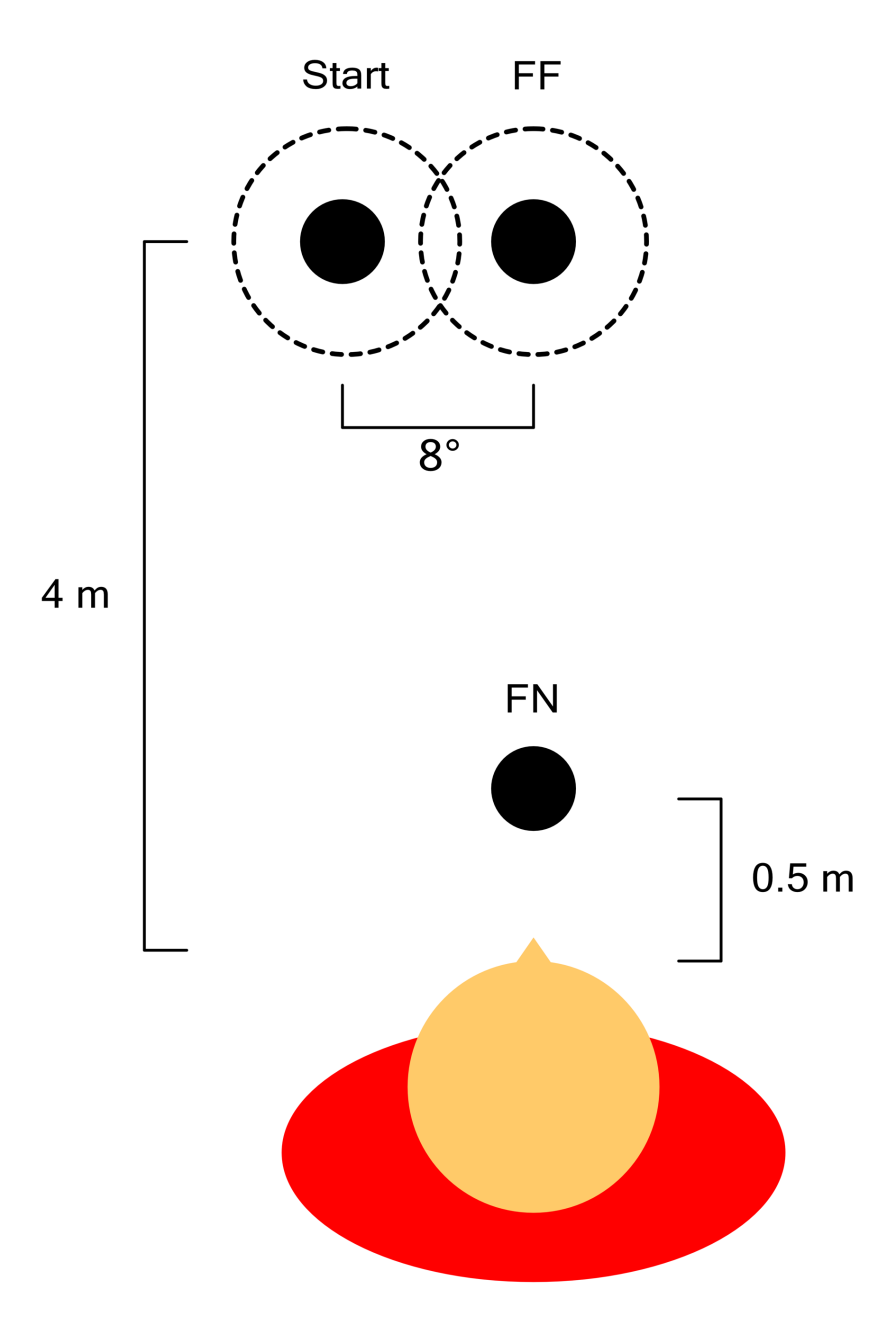
Schematic overview of experiment 1. Participants first fixated the target at a far location offset by 8° to the left (shown) or right. The target then either moved to another far position (FF) or a near position (FN), both at 0° azimuth. In half of the trials the size of the target was corrected to its distance from the participant so that it always occupied an equal area of the visual field (dotted lines).

In the first experiment, participants were asked to fixate a spherical target. The target first appeared at a distance of 4m at an offset of 6° azimuth either to the left (50% of the trials) or to the right. The target would remain at this location for 3 s to allow pupil size to stabilize after which it would jump to either of two positions, depending on the condition. In far-to-far (FF) trials the target remained at a 4 m distance, but jumped to 0° azimuth. In far-to-near (FN) trials the target would jump to a distance of 0.5 m and to 0° azimuth (see Figure 1 for a schematic overview). The target remained at the second location for 5 s, after which it disappeared for 1 s before the start of the next trial. In half of the trials, the target’s virtual diameter remained constant at 0.07 m (equal to an 8° visual angle at a distance of 0.5 m), covering a larger portion of the visual field when presented closer to the participant (non-corrected size). In the other half of the trials, the target’s diameter was modified to occupy a fixed 8° of the visual field, independent of target distance (corrected size). This means that the target was made larger when it was positioned at the far location. In the latter condition, any remaining minor differences in luminance between target and background could not be mediated by the size of the target relative to the visual field, as it was held constant. In summary, this experiment followed a fully crossed 2 (distance: FF vs. FN) x 2 (target size correction: non-corrected vs. corrected) design. Each of the resulting four conditions contained 12 trials each. However, the presentation order of trials was fully randomized.

Pupil data were recorded continuously by the built-in eye-tracker of the HTC Vive Pro Eye at roughly 90 Hz. Infrequent sampling was the result of sampling being coupled to the time it took the experiment to update a frame (i.e. to make all calculation to determine what should be shown in the next frame). Whenever a new trial started, a trigger was added to the pupil data which was used to cut the pupil recording into 12 eight-second segments (traces) per condition. As such, each trace corresponded to an individual trial. Traces represented the period from when the target appeared in the first location to when the target disappeared at the second location. During data collection, all time points where participants had their eyes closed were marked as part of a blink. If more than 20% of a trace consisted of marked values it was removed from further analysis. This occurred five times with a maximum of one occurrence per participant.

As the closing of the eyes can cause artefacts in the pupil data, all data within 67ms before the last marked value before blink-onset were considered to be part of the blink (22). Similarly, the opening of the eyes can cause artefacts and so the end of a blink was defined as the value following the first 133ms period without marked values. Data were resampled to 60 Hz and values corresponding to blinks were replaced through linear interpolation. The resulting traces were slightly smoothed to remove high-frequency noise by using an 11-point moving average filter. The jump of the target was considered as time point 0. Baseline pupil size (BPS) was defined as the average pupil size during the first 100ms after the target had jumped, before the pupil had time to react. To acquire an event-related response, each trace was baseline corrected by subtracting BPS from all values in the trace. After pre-processing, traces within a condition were averaged into one mean trace per condition, per participant. Next the pupil constriction amplitude (PCA) was calculated by taking the difference between the maximum value in the first 500ms after the target jump (0 – 500 ms) and the minimum value in the remainder of the trace (500 – 5000 ms). PCA was analysed as the dependent variable using a two-way repeated measures ANOVA with the aforementioned factors “distance” and “target size correction” as independent variables.

### Experiment 2

**Figure 2.**
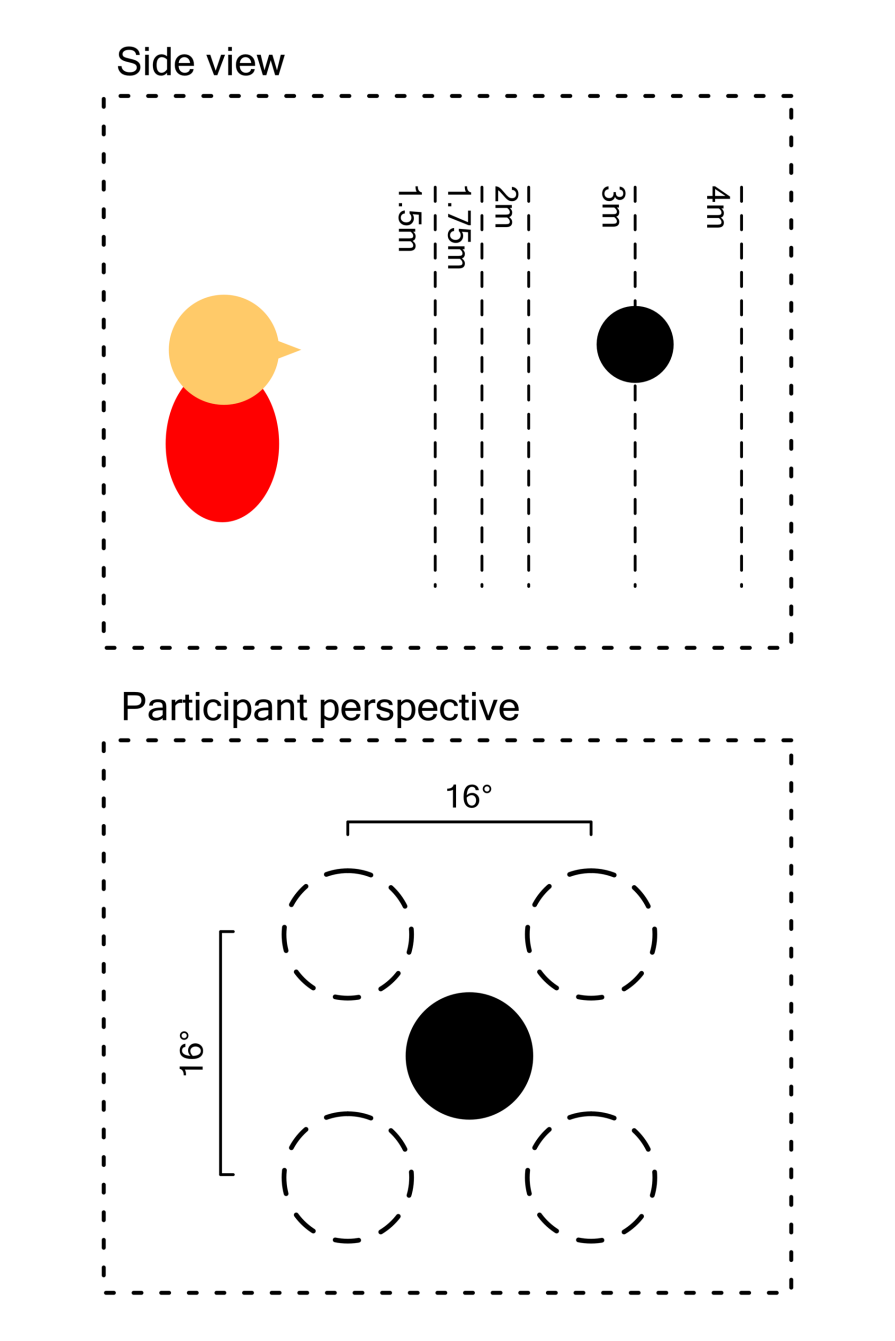
Schematic example of a trial in experiment 2. Dotted lines in the side view show all possible distances. Dotted circles in the participant perspective show alternative target positions at a certain distance.

Similar to the first experiment, participants were asked to fixate a spherical target. However, this time the target could appear at any of five locations, corresponding to the corners and centre of a square with a height and width of 16° of the visual field (Figure 2). Within a block, target distance was fixed and the target would appear on all five locations in random order, remaining at each location for 5 s. This was repeated for five blocks, each positioning the target at a different distance (1.5 m, 1.75 m, 2 m, 3 m and 4 m), as derived from Iskander, et al. (9). The order of the distances was randomized. Between blocks the target disappeared for one second. All blocks were presented twice, once where targets had a consistent size of 0.07m (non-corrected size), and once where their size was manipulated so that it corresponded to 8° of the visual field (corrected size), regardless of the target’s distance. All non-corrected and corrected blocks were clustered; participants randomly started with either the non-corrected or corrected ones. Pupil data corresponding to the five locations were averaged, resulting in a 5 (distances) x 2 (non-corrected vs. corrected) design.

For experiment 2, pupil data were cut into traces that corresponded to the period between the moment when the target had appeared at a certain distance and when it disappeared before moving to the next distance (25 s). Quality control and pre-processing was identical to that of experiment 1. This time no traces were excluded. All values within a trace were averaged so that each distance was represented by one average pupil size value. These values were analysed using a two-way repeated measures ANOVA.

As an additional post-hoc analysis, size corrected traces were separated into 5 second periods representing individual target fixations. However, to avoid overcomplicating the analysis, the corrected versus non-corrected comparison was omitted. Instead, only corrected conditions were included in the analysis, which better controlled for potential (subjective) luminance effects. Traces were baseline corrected by subtracting the average pupil size during the first 100 ms after the target had appeared or jumped. The first fixation after the target had changed distance was assigned to be either representing a far-to-near (FN) change (if the previous distance was further away) or a near-to-far (NF) change (if the previous distance was closer by). The remaining four fixations were averaged into one trace, which was assigned to represent no change in distance. Traces within a condition (no-change, far-to-near and near-to-far conditions) were averaged and PCA was calculated using the same method as experiment 1.

## Results

### Experiment 1

**Figure 3.**
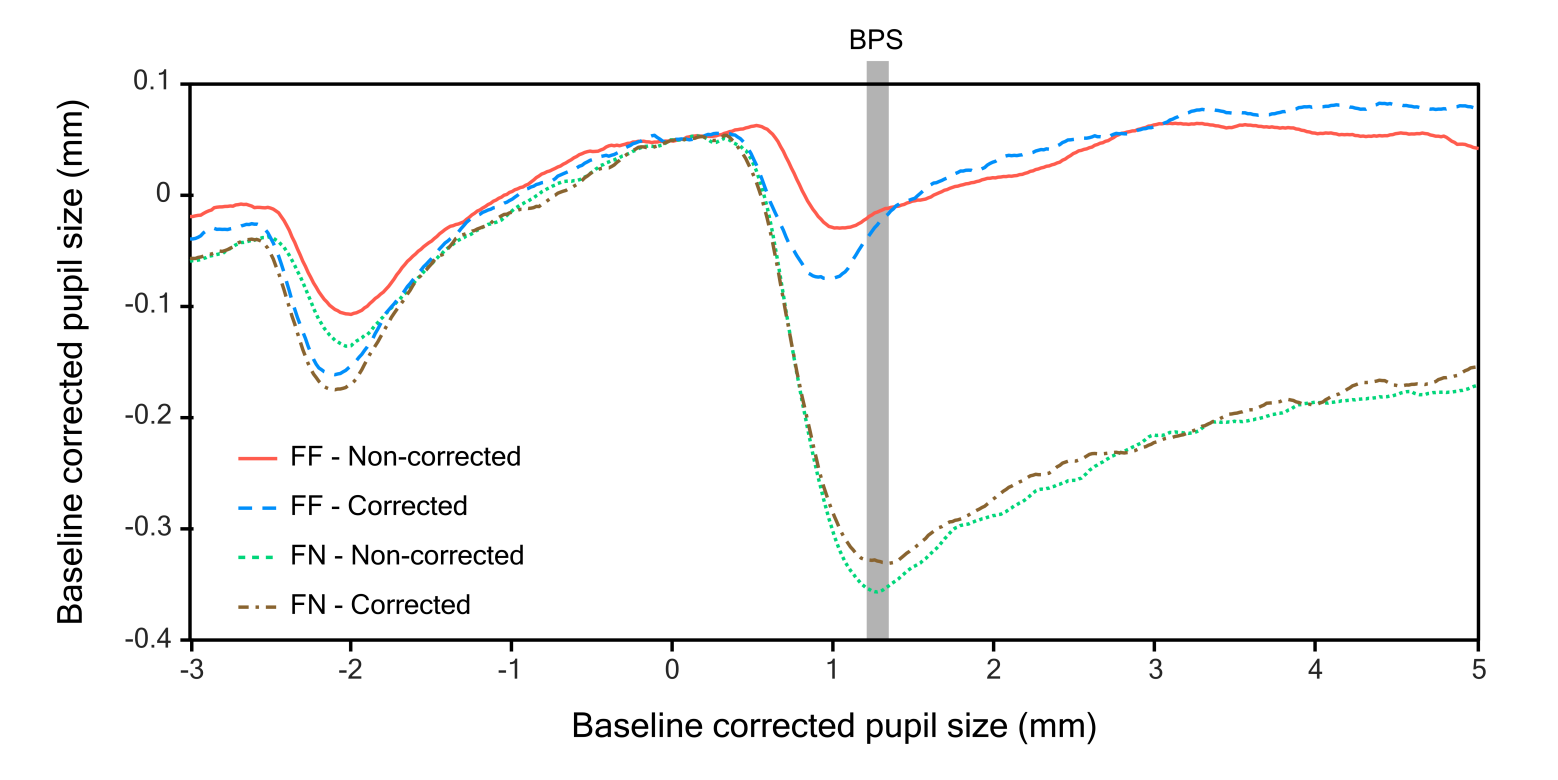
Grand mean pupil traces of experiment 1. At -3 s the target first appeared at a far position. At 0 s the target jumped to the second location. The period from 0 to 0.1 s, marked by the grey box, indicates the time period over which baseline pupil size (BPS) was calculated.

**Figure 4.**
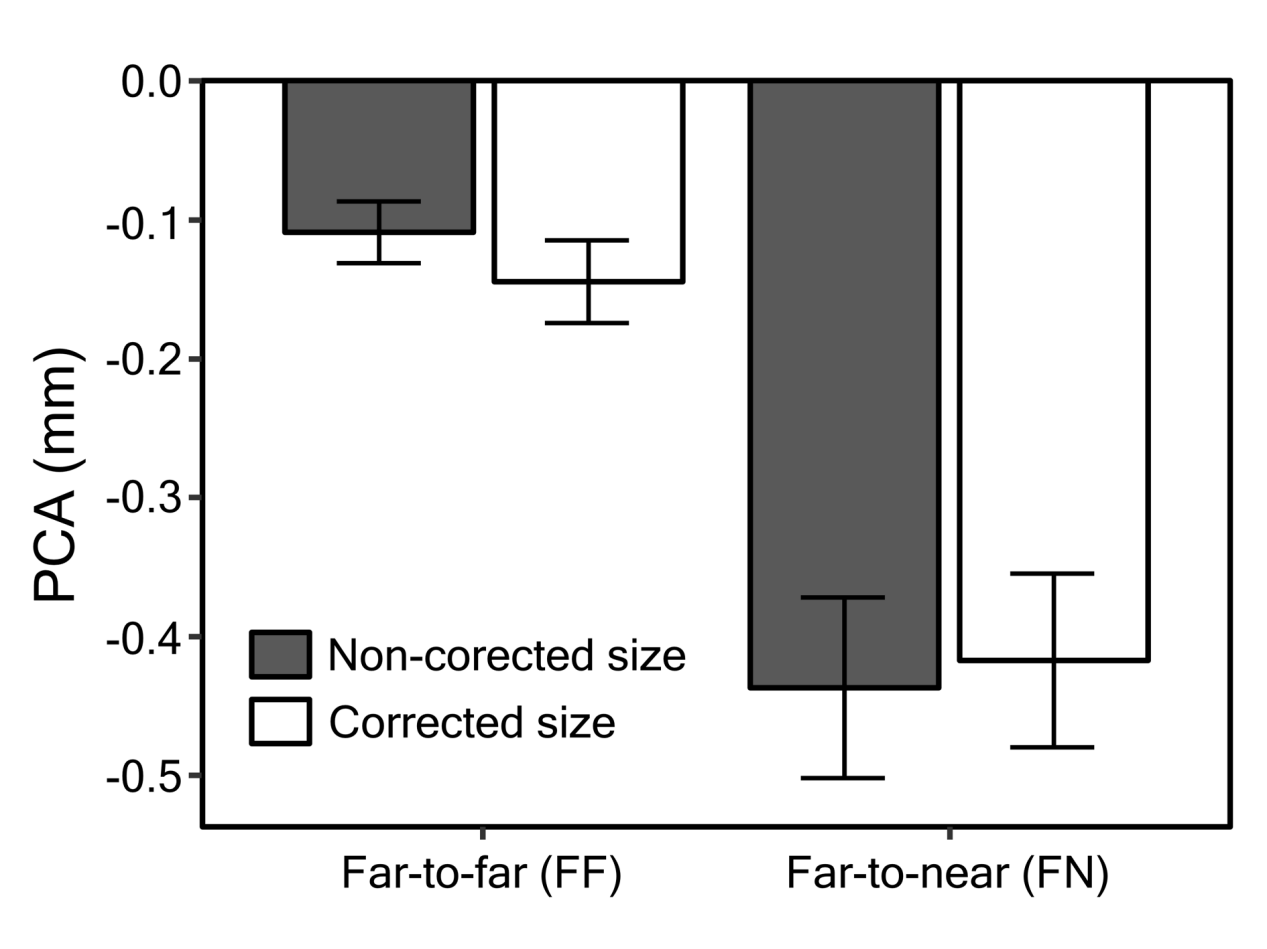
Mean pupil constriction amplitudes (PCAs) of experiment 1, together with standard errors.

Participants had a mean BPS of 3.38 mm (SD = 0.60) across all conditions. Grand mean traces were plotted to visualize the task evoked pupils responses (Figure 3). All traces show a constriction whenever participants fixated the target, either at the first location or the second one. A trend is visible where FN conditions caused a more pronounced constriction of the pupil than FF conditions. Similar observations were made for the bar plots of PCA data across conditions (Figure 4). Indeed, the ANOVA revealed a significant main effect of PCA for FF vs. FN, F(1,28) = 126.86, p < .001, meaning that the pupil near response was expressed most strongly when targets jumped nearer. While there was no main effect for target size correction, F(1,28) = 0.61, p = .440, a significant interaction between target size correction and distance was found, F(1,28) = 8.56, p = .007. Figures 3 and 4 show that the FF constriction was slightly greater and the FN constriction was slightly smaller for trials where target size was corrected, compared to trials where it was not. Post-hoc t-tests (Bonferonni correction applied) did not find a significant difference between non-corrected and corrected FN trials, t(28) = -1.36, p = .369. However, FF trials were found to differ between size correction conditions, t(28) = 2.69, p = .024. This means that the pupil constricted more strongly when large, rather than small, targets moved from a far position to another far position.

### Experiment 2

**Figure 5.**
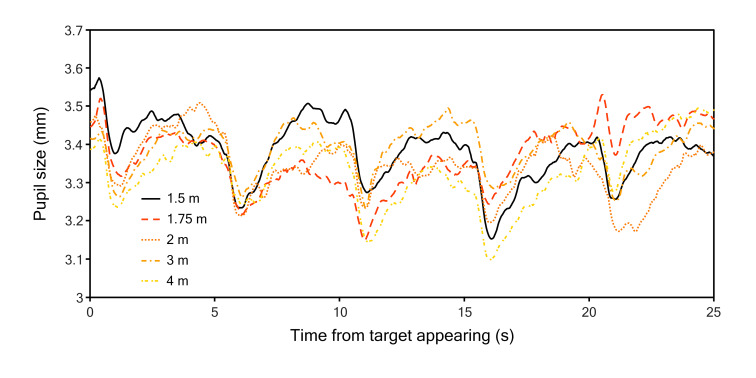
Full 25 second pupil traces of experiment 2. The target first appeared at 0 s. Every five second the target jumped to the next location.

**Figure 6.**
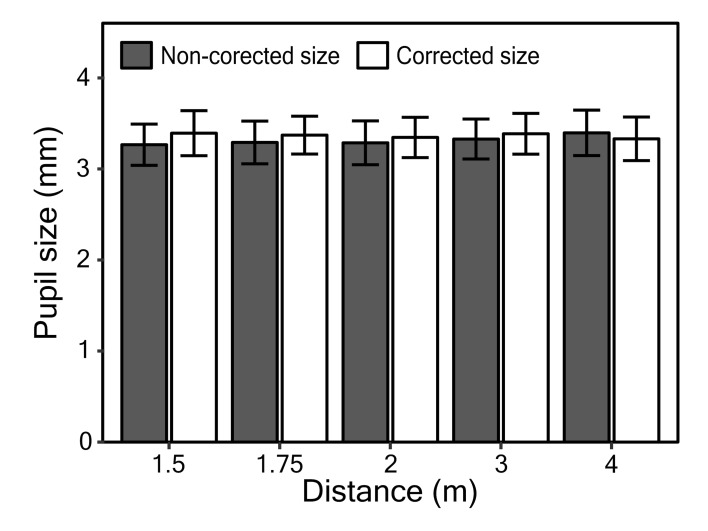
Average pupil sizes of experiment 2, together with standard errors. The target first appeared at 0 s. Every five second the target jumped to the next location.

In experiment 2, participants had a mean BPS of 3.43 mm (SD = 0.67) across all conditions. These BPS values did not significantly differ from those found in experiment 1, t(28) = -1.42, p = .167. The full 25 s traces of each distance have been plotted in Figure 5. There is a clear pupil constriction after each time the target would move, possibly the result of an orienting response (25). However, overall there are no discernible trends in the pupil data between depth conditions. Plots of the average pupil size data from experiment 2 can be found in Figure 6. The data showed no influences of distance or target size correction on average pupil size. Indeed, analysing the data using an ANOVA did not reveal a main effect of distance, F(4,112) = 0.61, p = .658, or target size correction, F(1,28) = 2.97, p = .096. Nor was there an interaction effect, F(4,112) = 1.60, p = .181).

**Figure 7.**
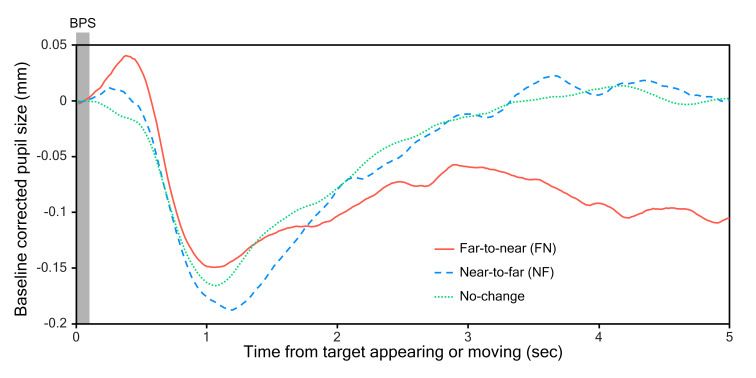
Grand mean pupil traces of 5 second target presentations in experiment 2. At 0 s targets either appeared (after a distance switch) or jumped to a different location at the same distance. The period from 0 to 0.1 s, marked by the grey box, indicates the time period over which baseline pupil size (BPS) was calculated.

For the additional post-hoc analysis, pupil data were cut to 5 s traces representing the presentation of the target at each location and distance. These traces were ordered based on the condition whether there was no distance change, a NF change or a FN change. Traces within each condition were averaged across trials and can be found in Figure 7. PCA calculated from these traces was found to differ significantly between the no-change, FN and NF conditions, F(2,56) = 5.95, p = .005. Post-hoc t-tests (Bonferroni corrected) revealed that PCAs in the FN and NF conditions significantly differed from PCAs in the no-change condition with t(28) = -3.94, p = .001 and t(28) = -2.72, p = .033 respectively. This means that any change in distance caused a greater constriction than no change. However, the FN and NF conditions did not significantly differ from each other, t(28) = -0.56, p = 1.000, suggesting that it does not matter if the distance change is positive or negative. Figure 7 shows that in the period between 2 and 5 s after the target had moved, the FN trace differs a lot from the FN and no-change traces. However, when comparing average pupil size during this period, no significant effect was found F(2,56) = 0.84, p = .437. Likely, as there were very few FN and NF trials per participant, not enough traces were available to account for random noise in the signal and properly capture the pupil response. The resulting noisy traces could have introduced a lot of between-subject variation in the pupil size average. Indeed, the average pupil size measures of both the FN (mean = -0.08, SD = 0.24) and NF (mean = -0.01, SD = 0.32) conditions had very large standard deviations compared to the NC condition (mean = -0.01, SD = 0.06), which included much more traces.

To further explore the data, the first 5 s of pupil data after a change in distance were organized into different conditions, based on the distance of the previous and current trial. Traces in each condition were averaged across participants and PCA values were calculated. These values can be found in Table 1. The table reveals no clear pattern that indicates different magnitudes of pupil constrictions for FN trials and/or pupil dilation for NF trials.

**Table 1.** Mean PCAs for Every Change in Distance.

		**From distance (m)**				
		**1.5**	**1.75**	**2**	**3**	**4**
**To distance (m)**	**1.5**	X	-0.47	-0.53	-0.45	-0.42
	**1.75**	-0.39	X	-0.44	-0.43	-0.50
	**2**	-0.41	-0.33	X	-0.72	-0.36
	**3**	-0.29	-0.55	-0.29	X	-0.38
	**4**	-0.68	-0.49	-0.48	-0.21	X

Note. Columns represent the distance of the target on the previous trial. Rows represent the distance the target jumped to. Since PCAs were only calculated whenever the target changed its distance, cells representing no change have been marked with an ‘X’.

## Discussion

Experiment 1 assessed whether a gaze shift from far-to-near (FN) would cause a greater pupil constriction response as compared to a gaze shift from far-to-far (FF). Indeed, we found that the amplitude of the pupil constriction in response to a target jump was greater following FN trials, compared to FF trials. This is in line with pupil behaviour found outside of HMD-mediated VR (5; 7; 11) and implies that the pupil near response is intact in HMD-mediated VR, regardless of the vergence-accommodation conflict. As accommodative changes are mostly absent in HMD-mediated VR (8), this finding lends further support to the idea that the pupil near response is mostly coupled to vergence (5).

The FF vs. FN manipulation was found to interact with target size correction; when target size was corrected, FF constriction was found to be more pronounced. The pupil constriction found in FF trials was unlikely to be related to the pupil near response (as the target did not come nearer). Instead, it could have reflected a pupil orienting response, which has been found to scale with salience (25; 26). As targets were larger in non-corrected (compared to corrected) trials, they appeared more salient. This could explain why non-corrected FF constrictions were more pronounced. Alternatively, the interaction of FF vs. FN trials with target size correction could be partially explained by the fact that the corrected FN condition lacked the natural change in target size. Such an unexpected event (people expect the target to become bigger, in line with the vergence signal) may evoke a dilatory surprise component on top of the pupil near response (12; 14; 19).

In experiment 2, participants fixated a target that appeared at several locations and would sometimes change its distance. It was assessed if average pupil size during target fixations differed depending on the distance of the target. The results suggest this was not the case. Possibly, the pupil is sensitive to transient and substantial changes in stimulus distance only. There might not be a direct relationship between target distance and pupil size (under equiluminant conditions) when observers fixate targets that are repeatedly displaced with only occasional changes in distance. This implies that the pupil near response does not result in a sustained change of pupil size. This contradicts the findings by Iskander, et al. (9), who found increased pupil size when targets appeared closer by. It was theorized that these previous findings could be explained by (subjective) differences in target luminance caused by variations in the target’s size relative to the visual field. However, even in trials where target sizes were non-corrected, we could not replicate these findings.

If pupils are only sensitive to transient and substantial changes in stimulus distance, experiment 2 should also have caused a pupil response whenever the target jumped to a different distance. While the pupil was found to show a greater PCA whenever the target moved to a nearer distance, compared to when it remained at the same distance, the same was found for trials where the target moved further away. This is counter intuitive, as near-to-far events should have evoked pupil dilations, or at least weakened pupil constrictions. It is possible that these findings are the result of the one second interval between changes in distance, during which the target was absent. The reappearance of the target after this second could have caused an initial dilatory surprise effect (19), somewhat suppressing a distance-induced constriction. This effect would then have been absent whenever the target moved without changing its distance (and thus did not dis- and reappear). This is somewhat reflected in Figure 7, implying that the FN and NF trials caused a pupil dilation shortly after the target appearing. The same dilation is not present in the no-change condition. As an alternative explanation, the temporary disappearance may have caused a lacking or diminished re-orienting response (people lost track of, or interest in, the target), effectively disrupting the NF and FN events. Indeed, the PCAs in experiment 2 were overall weaker than in experiment 1 (compare axis of Figure 3 and 7). This would also explain why there is no detectable pattern between PCA and the direction of distance change magnitude, as reflected in Table 1. It is recommended for future studies using similar designs to avoid including periods where the target is absent to avoid that this can influence the pupil near response.

A limitation of this experiment is the fact that all distance cues (e.g. shadows and relative target size in half of the trials) were removed from the targets. Previous research has found that stimuli that are lacking in distance cues do not properly stimulate accommodation (18). While accommodation in HMD-mediated VR is disturbed regardless of distance cues (8), the lack of distance cues could have elicited accommodative responses which differ from accommodative responses that normally occur in HMD users. Possibly, with more distance cues intact, accommodation could have an influence on the pupil near response. Regardless, it is clear that vergence is sufficient for the pupil near response to occur. Future studies should consider including conditions with distance cues intact. Furthermore, the design could be complemented by including EOG measurements, which inform about vergence (21). This would allow for deeper insights in how vergence and pupil size are related.

## Conclusion

When shifting gaze between a far target and a near target in HMD-mediated VR, the pupil constricts as it would outside of VR. This provides evidence that the vergence-accommodation conflict caused by HMDs does not disrupt the pupil near response. Furthermore, as HMDs are known to disrupt accommodation, the findings from this study support the idea that the pupil near response is mostly coupled to vergence. No evidence was found to suggest that stimulus distance in HMD mediated VR affected average pupil size when fixating gaze on that stimulus.

### Ethics and Conflict of Interest

The authors declare that this project was in line with the ethics described in https://bop.unibe.ch/JEMR/about. The authors declare no conflicts of interest.

### Acknowledgements

This project has received funding from the European Union’s Horizon 2020 research and innovation programme under the Marie-Sklodowska-Curie grant agreement No 765329.
